# Experimental Investigation on a Novel Temperature-Controlled Phase Change Aggregate Concrete: Thermo-Mechanical Properties and Hydration Heat Control

**DOI:** 10.3390/ma16155269

**Published:** 2023-07-27

**Authors:** Yejia Wang, Chengjin Wang, Aibo Luo, Minqi Dong, Qian Su, Chenling Zhou, Zongyu Zhang, Yanfei Pei

**Affiliations:** 1 Department of Civil and Environmental Engineering, Imperial College London, London SW7 2AZ, UK; wyjic1211@163.com; 2 School of Civil Engineering, Southwest Jiaotong University, Chengdu 610031, China; wangchengjin2023@163.com (C.W.); luoaibo112@hotmail.com (A.L.); swjtuzcl@outlook.com (M.D.); xnjd98@126.com (Q.S.); zcl@my.swjtu.edu.cn (C.Z.); 13608101631@163.com (Z.Z.)

**Keywords:** mass concrete construction, hydration heat control, phase change aggregate, phase change concrete, thermo-mechanical properties

## Abstract

To reduce the structural deterioration of mass concrete structures from temperature cracks, and lower energy consumption caused by the traditional mass concrete hydration heat cooling process, this paper reports the preparation of concrete temperature-controlled phase change aggregate (PCA) by a vacuum compaction method using light and high-strength black ceramite and No. 58 fully refined paraffin wax as phase change material (PCM), and the encapsulation technology of the aggregate by using superfine cement and epoxy resin. Further, through laboratory tests, the cylinder compressive strength, thermal stability and mixing breakage rate of the encapsulated PCA were tested, and the differences in mechanical properties such as compressive strength, flexural strength and splitting tensile strength between phase change aggregate concrete (PCAC) and ordinary concrete were studied. A test method was designed to test the heat storage effect of PCA, and the temperature control effect of PCAC was analyzed based on the law of conservation of energy. The research conclusions are as follows: (1) Both superfine cement and epoxy resin shells increase the strength of the aggregate, with the epoxy resin increasing it more than the superfine cement. The thermal stabilization of the PCA is good after encapsulation of superfine cement and epoxy resin. However, PCA encapsulated in superfine cement is more easily crushed than that encapsulated in epoxy resin. (2) Under the condition of water bath heating and semi-insulation, when the water bath temperature reaches 85 °C, the temperature difference between the PCA and the common stone aggregate can be up to 6 °C. Based on the law of energy conservation, the test results will be converted to mass concrete with the same volume of aggregate mixture;, the difference of PCAC and ordinary concrete temperature can be up to 10 °C, so the temperature control effect is significant. (3) The mechanical properties of PCAC with 100% aggregate replacement rate compared to ordinary concrete are reduced to varying degrees, and the performance decline of the epoxy-encapsulated PCA is smaller than that encapsulated with superfine cement; in an actual project, it is possible to improve the concrete grade to make up for this defect.

## 1. Introduction

With the development of human society, global resource and energy scarcity have become a challenge that mankind has to face. Civil engineering construction is an area of great resource and energy consumption. Infrastructure construction and maintenance consume resources and energy and cause pollution. It is estimated that about 70% of global greenhouse gas emissions come from infrastructure construction and operation [1]. Therefore, modern infrastructure is now needed to meet the growing demand for enhanced sustainability, green economy, smart performance and extended service life [2].

**Figure 1:**
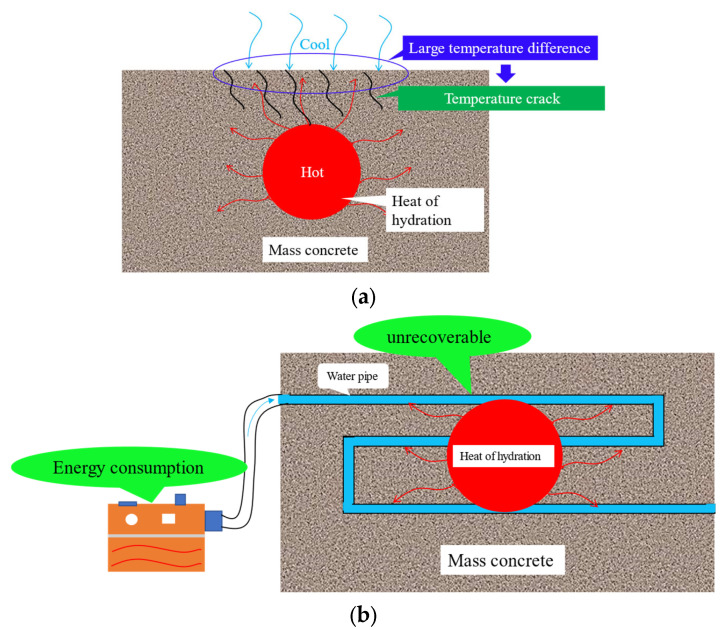
The risk of heat of hydration in mass concrete and traditional control techniques. (**a**) Principles of mass concrete cracking due to heat of hydration. (**b**) Technology for temperature control of heat of hydration in mass concrete based on condensation tubes.

Mass concrete is widely used in large modern buildings due to its excellent integrity and stability [3,4,5,6]. However, in the actual engineering, cracks will occur on the surface due to the large amount of heat generated from the hydration of cement inside mass concrete, as shown in Figure 1a. Statistical studies show that temperature cracks of mass concrete account for about 80% of the total cracks of engineering structures [7]. Usually, in order to avoid concrete cracking, cooling water pipes are arranged in the mass concrete. However, the pipes arranged in this way will not be recycled, and the cooling water circulation also needs to consume a lot of resources and energy as shown in Figure 1b.

In recent years, the use of PCM to control the heat of hydration of mass concrete is a new temperature control method. The mechanism of this technique is to reduce the temperature difference between the inside and outside of the mass concrete by absorbing the heat of hydration and slowing down the cooling rate of the mass concrete through the phase change of the material, thus reducing the internal temperature stress of the mass concrete [8]. PCM can be classified into organic PCM, inorganic PCM and eutectic mixtures according to their material composition. Sharma, A et al. [8] classified organic-type PCM into paraffinic and non-paraffinic compounds (e.g., fatty acids), and inorganic PCM into two categories: hydrated salts and metal compounds. When the heat of hydration of mass concrete is controlled by PCM, there are various blending and packaging methods, which can be summarized into four categories: PCM is adsorbed by lightweight aggregate [9,10,11,12,13]; PCM is filled in the pipeline [14,15,16,17,18]; PCM is coated with microcapsules [19,20,21,22]; PCM is immersed through cracks in the concrete surface [23,24,25].

The idea of using lightweight aggregate to adsorb PCM comes from mixing water stored in PCA into concrete to prevent its self-shrinkage [9,10,11], and it has a similar application in self-healing concrete [12,13]. Therefore, many scholars have applied the same idea in the preparation of PCA, and have carried out related studies on the mechanical and thermal properties of PCAC mixed with PCA. Different kinds of lightweight aggregates are used in the preparation of PCA. Expansive clay, as a lightweight aggregate that can be mixed in concrete, is often used as a PCM carrier. Nepomuceno and Sakulich [26,27] incorporated expanded clay into cement mortar as a PCA, and found that the thermal response of mortar increased with the increase in PCM, but the mechanical properties were affected. Shazim and Sakulich [28,29] incorporated expanded clay into concrete as a PCA, indicating that PCA has good temperature control and excellent mechanical properties. The literature [30,31,32,33] used expanded shale as a light aggregate to adsorb PCM and incorporated it into concrete; the study found that the PCA had a high adsorption rate for PCM and a significant energy storage effect of PCAC, and analyzed the thermal conductivity of the PCA. Many scholars had also used perlite [34,35] and diatomite [36,37,38,39] as PCA to prepare PCAC, the test results showed that PCA had high thermal stability and adsorption rate, and PCAC had good thermal storage and temperature control effect, but the mechanical properties of PCAC, such as compressive strength and flexural strength, had decreased to a certain extent.

Compared with the embedded pipe method, the use of PCA can better disperse the PCM in every corner of the concrete, and the temperature control effect is more uniform. However, relevant studies have shown that if the PCA adsorbed with the PCM is directly added to the concrete, the mechanical properties of the concrete will be sharply reduced. The main reason is that the phase change material flows out of the aggregate after the phase change [40,41], and mixes with the cement slurry, hindering the hydration reaction of the cement in the concrete. Leakage of PCM can lead to a weakening of the bond between cementite materials and coarse aggregate [42], thus affecting concrete strength. Therefore, it is necessary to build a hard shell on the outer layer of PCA.

Phase change temperature control technology has the advantages of a uniform temperature control effect and little influence from construction factors, which provides a new solution for mass concrete hydration heat temperature control. Now, the technology of controlling the hydration heat of mass concrete with PCM is still in its infancy and has not been widely used in practical engineering. Based on the hydration heat control technology of mass concrete, this study prepared a structure-function integrated PCA, carried out its performance test, mixed the PCA into the concrete to study the physical and mechanical properties of the PCAC and finally designed a heat storage test method to study the heat storage effect of the PCAC. It will provide a valuable reference for the popularization and application of PCAC in practical engineering.

## 2. Materials and Equipment

### 2.1. Aggregate

**Figure 2:**
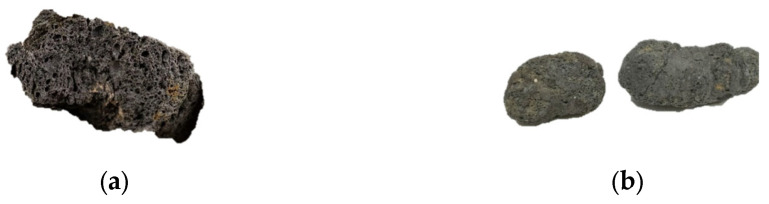
Physical appearance of lightweight black ceramic granules. (**a**) Profile of ceramic granules. (**b**) Geometry of lightweight black ceramic granules.

In this study, lightweight ceramic granules are selected as the aggregate. Light-weight ceramic granules are porous inside, have high air permeability, water permeability and high strength. Its main performance indexes are shown in Table 1, and its physical appearance is shown in Figure 2.

### 2.2. Phase Change Materials

**Figure 3:**
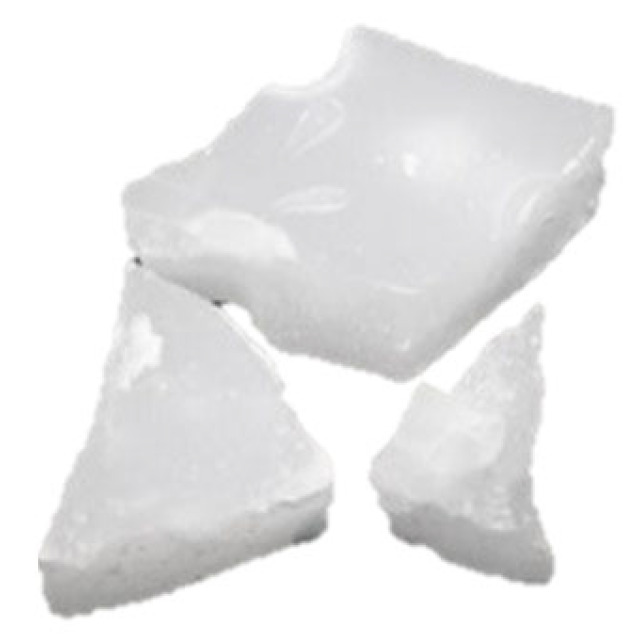
No. 58 fully refined paraffin indoor physical display.

The PCM used in this study is No. 58 fully refined paraffin, which is a white block, and the purity of the paraffin used is high. The basic performance indexes of paraffin are shown in Table 2, and the physical display is shown in Figure 3.

### 2.3. Encapsulating Materials of PCA

To avoid the liquid paraffin flowing out of the PCA after the temperature of the concrete increases, it is necessary to conduct a shell-forming encapsulation of the surface of the PCA, and wrap the PCM inside the PCA. In this study, superfine cement and epoxy resin were selected to encapsulate the PCA.

#### 2.3.1. Superfine Cement

This study selected 800 mesh superfine cement and its mechanical performance indicators are shown in Table 3.

**Figure 4:**
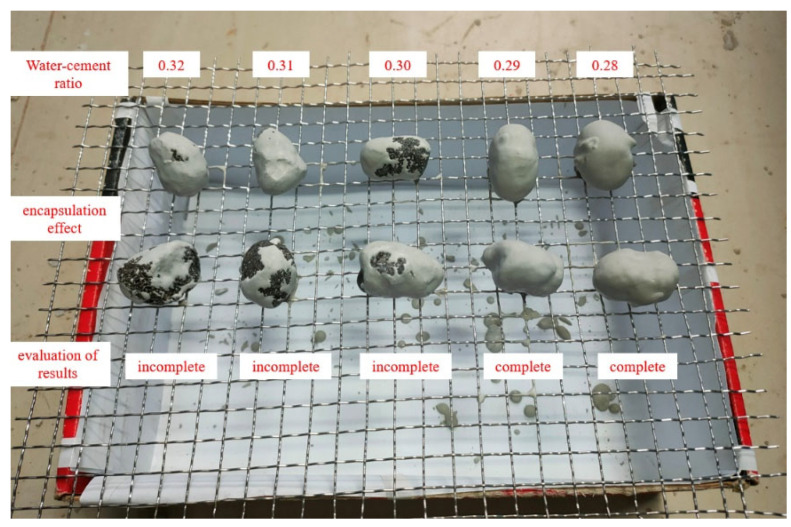
Results of superfine cement shelling test under different water–cement ratios.

In order to obtain the optimal water–cement ratio, superfine cement slurries with water–cement ratios of 0.32, 0.31, 0.30, 0.29 and 0.28 were configured, and the PCA encapsulation test was carried out by using cement slurries with each water–cement ratio. It was found that when the water–cement ratio was less than 0.29, superfine cement can completely coat the aggregate, as is shown in Figure 4. Therefore, a water–cement ratio of 0.29 was selected as the optimum water–cement ratio.

#### 2.3.2. Epoxy Resin

**Figure 5:**
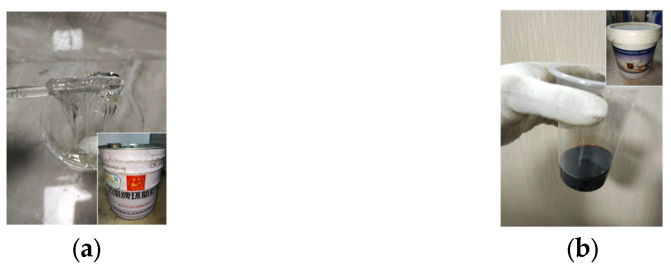
Physical diagram of components A and B of epoxy resin. (**a**) Component A; (**b**) Component B.

The second packaging material for the phase change aggregate used in this study is an epoxy resin, which is composed of two component raw materials (commonly known as A and B materials) prepared, stirred, reacted and solidified in a certain ratio. This study used bisphenol A-type epoxy resin, which was material A (raw material) and material B (curing agent) in a 2:1 ratio. After multiple indoor adjustments, the model of material A was determined to be WSR6101 (E44). The material is colorless and transparent, and its performance indexes are shown in Table 4. Material B is a special modified amine curing agent, which is a black liquid. The indoor physical display of raw materials A and B is shown in Figure 5.

To ensure the encapsulation quality of epoxy resin mixtures on aggregates, the following three necessary conditions must be met: (A) appropriate viscosity, (B) the operational time is longer than 20 min and (C) excellent thermal conductivity. Immerse the PCA into the epoxy resin to evenly adhere to its surface; the epoxy resin shell should completely cover the PCA, with a very smooth surface, uniform thickness and a light-yellow transparent shape. According to *Methods of viscosity measurement* (GB/T 10247–2008) [43] in the testing standards in China, the viscosity of epoxy resin was tested using a viscometer, and the viscosity of the epoxy resin was 2.48 Pa·s. The epoxy resin used meets the requirements for the viscosity of PCA shell materials. In this study, the gelling time of epoxy resin was tested by the gelling time tester, and the gelling time of epoxy resin was measured to be 27 min at 19 °C. The operational time of the epoxy resin prepared in this study meets the requirements for PCA encapsulation.

**Figure 6:**
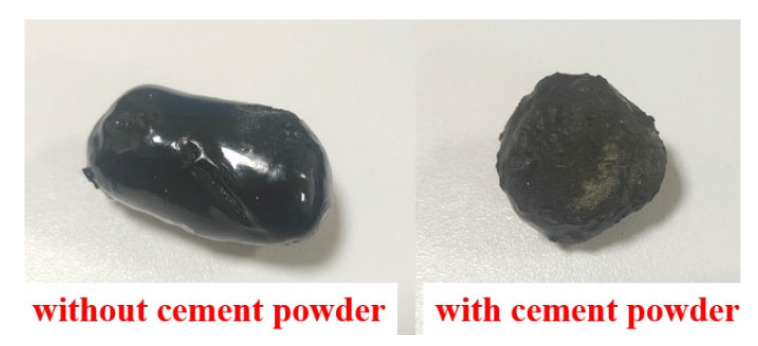
Physical image of epoxy resin PCA before and after wrapping with cement powder.

Shazim et al. [28,42] showed that the thermal conductivity of epoxy resin increased linearly with the amount of graphite powder added, The relationship can be described as *y* = 0.0202*x* + 0.2672, *R^2^* = 0.94, where *y* is the thermal conductivity, *x* is the graphite powder content, and *R* is the correlation coefficient. To increase the thermal conductivity of epoxy resin, a 15% graphite powder mixed with epoxy resin was used to shell the PCA. The aggregate with epoxy resin was wrapped in cement powder to increase the bonding effect between the aggregate and concrete. The indoor shell material display is shown in Figure 6.

### 2.4. Equipment

**Figure 7:**
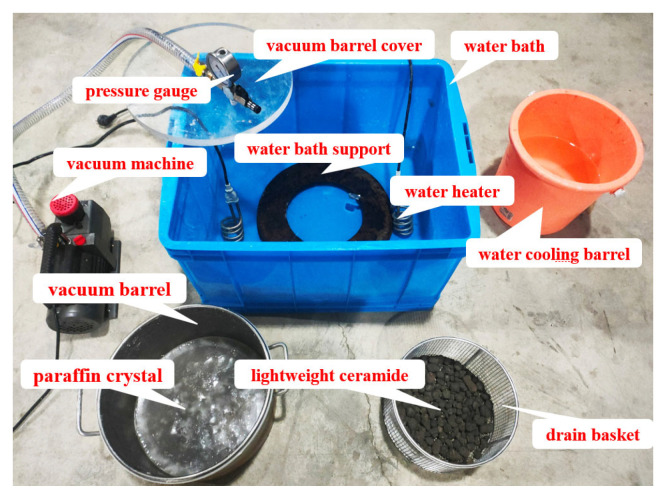
Self-made phase change aggregate preparation equipment.

Self-made equipment was used to prepare PCA indoors, as shown in Figure 7. The PCA preparation equipment includes PCM adsorption equipment and PCA water cooling equipment. The PCM adsorption equipment includes a water bath, a water bath support, a water heater, a vacuum barrel, a vacuum barrel cover, a drain basket and a vacuum machine. The phase change aggregate water cooling equipment is a water-cooling bucket.

## 3. Phase Change Aggregate Test Procedure

### 3.1. Preparation and Encapsulation Test of Phase Change Aggregate

Newly purchased ceramsites are not completely dry. Therefore, it is necessary to dry the ceramsites and remove surface shells from the dried ceramsites through a sieve shaker. Place the paraffin crystals into a vacuum bucket and melt them using a hot water bath, add ceramsites to the molten paraffin and start vacuum adsorption. All ceramsites must be 2 cm below the molten paraffin liquid level. The vacuum adsorption of PCM lasts for 60 min. After the adsorption is completed, remove the vacuum equipment, lift the drain basket and quickly pour the ceramsites that adsorb paraffin into the water-cooled bucket to avoid liquid paraffin flowing out of the ceramsites as much as possible. After water cooling, there is a trace of water on the surface of the PCA, which needs to be dried in a natural environment to evaporate surface moisture.

**Figure 8:**
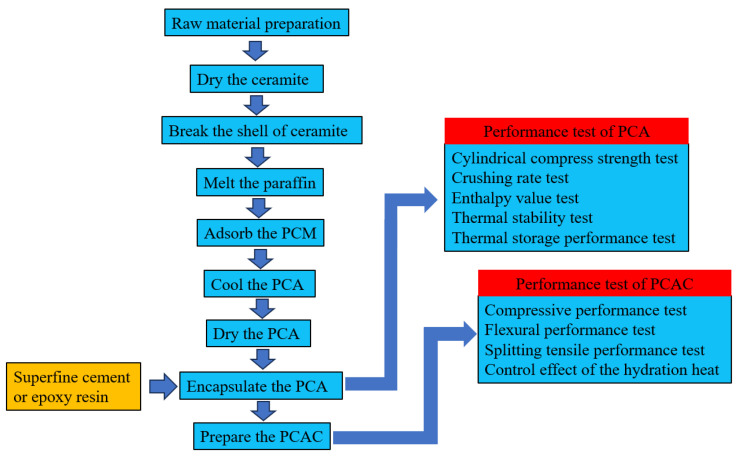
Flowchart of preparation and testing of PCA and PCAC.

The encapsulation operation of PCA using superfine cement slurry and epoxy resin is similar. The PCA granules are immersed in a freshly mixed superfine cement slurry/epoxy resin, and then thoroughly stirred to ensure that enough encapsulation material adheres to the surface of the PCA. Finally, the PCA granules are dried and formed. If there are still defects in the encapsulation effect, the phase change aggregate can be re-encapsulated. The preparation and performance test flow charts of PCA and PCAC are shown in Figure 8.

### 3.2. Mechanical Properties of PCA

#### 3.2.1. Cylindrical Compress Strength of PCA

**Figure 9:**
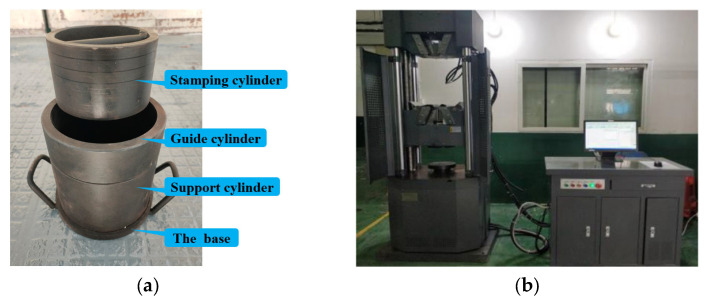
Cylinder compressive strength test of PCA. (**a**) Pressure cylinder; (**b**) Universal testing machine.

Conduct cylindrical compress strength tests on ceramsites, unencapsulated PCA, superfine cement-encapsulated PCA and epoxy resin-encapsulated PCA, according to the lightweight aggregates and its test methods according to the Chinese Standard (GB/T17431–2010) [44]. The cylinder compression strength test instrument used for the PCA is shown in Figure 9.

Load at a constant speed of 300~500 N, and record the pressure value when the stamping depth is 20 mm. The cylinder pressure strength of each material is calculated according to the formula:

(1) $$f_{a}=\frac{p_{1}+p_{2}}{F}$$

where *f_a_* is the compressive strength of the coarse aggregate cylinder, and the unit is MPa. *p*_1_ is the pressure value when the pressing depth is 20 mm, the unit is Newton (N); *p*_2_ is the mass of the stamping cylinder, the unit is Newton (N), and the mass of the stamping cylinder in this study is 1.85 kg; *F* is the pressure area (in this study, *F* is 10,000 mm^2^). The cylinder pressure strength is the mean value of the three measurements. If the difference between the maximum value and the minimum value of the three measurements is only greater than 15% of the mean value, the sample should be retested.

#### 3.2.2. Crushing Rate of PCA during Mixing

To study the defects caused by the collision of PCA with other coarse aggregates, the PCA granules that have completed the shell-making process and coarse aggregates are directly added to the forced mixer for mixing to simulate the most severe environment of PCA during the mixing process. Mix 100 superfine cement-encapsulated PCA slurry with 20 kg coarse aggregates and place them in a forced mixer for mixing. The mixing time is 240 s, which meets the requirements of the Standard for quality control of concrete (GB50164–2011) [45]. The same operation is adopted for PCA encapsulated with epoxy resin. After mixing, separate the PCA from the coarse aggregates, observe the integrity of each PCA shell, and calculate the PCA shell crushing rate by Equation (2):

(2) $$P=\frac{n_{p}}{n}\times 100\%$$

where P is the crushing rate of the PCA shell, $n_{p}$ is the number of PCA with broken shells and n is the total number of PCA granules.

#### 3.2.3. Enthalpy Value of PCA

The phase-change temperature and latent heat of paraffin and PCA were measured by differential scanning calorimetry (DSC). DSC is to measure the change of the energy difference between the sample and the reference sample with temperature and time under the control of the setting temperature. A total of four sets of samples were selected for testing in this study, including two sets of paraffin samples and two sets of PCA samples. The four groups of samples are put into the DSC equipment for heating and cooling tests at 80 °C.

#### 3.2.4. Thermal Stability Test of PCA

**Figure 10:**
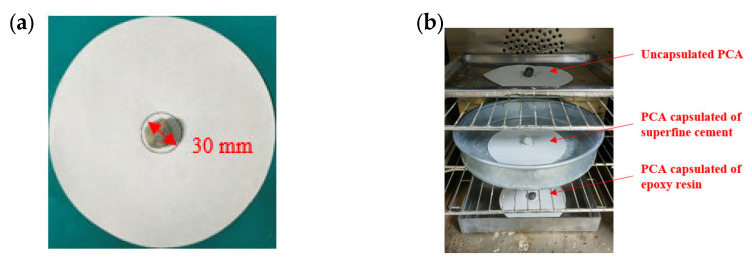
Thermal stability test of PCA. (**a**)The filter paper and PCA; (**b**) The electric blast drying oven.

To investigate the potential flow of PCM through micropores in the shell of PCA after complete melting, a thermal stability test was conducted using a heating-diffusion-exudation cycle method in this study. The following steps were taken during testing:

(1) Draw a circle with a diameter of 30 mm in the center of the filter paper, and place the PCA in the drawing area, as shown in Figure 10a.
(2) Place the PCAs to be tested in the electric blast drying oven and heat then at 100 °C for 24 h. The unencapsulated PCA sample is placed on the top layer of the oven, the PCA encapsulated in superfine cement is in the middle layer and the PCA encapsulated in epoxy resin is placed on the bottom layer, as is shown in Figure 10b.
(3) Observe the diffusion degree of the PCM on the filter paper, then measure the maximum diameter $d_{max}$ and minimum diameter $d_{min}$ of the phase-change material diffusion and calculate the mean $d_{0}$ of the maximum and minimum values.
(4) Calculate the percentage of exudation φ by Equation (3) as follows:
  (3) $$\varphi =\frac{d_{0}-30}{30}\times 100\%$$

#### 3.2.5. Thermal Storage Performance Test of PCA

**Figure 11:**
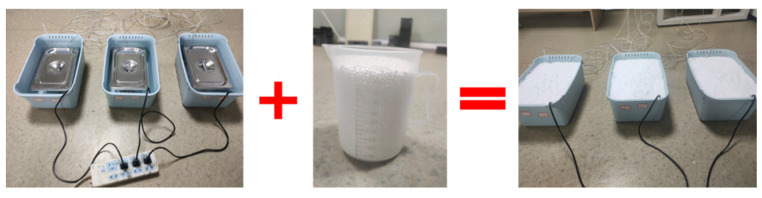
Add EPC insulation particles.

**Figure 12:**
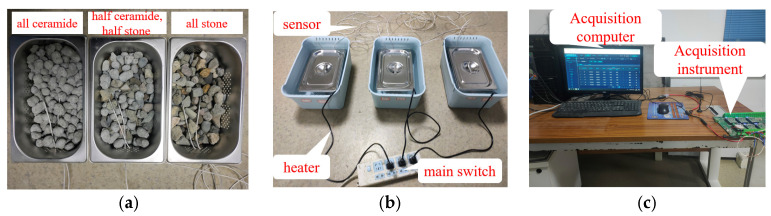
The experimental equipment. (**a**) Embed sensors; (**b**) Test equipment; (**c**) Collecting devices.

To investigate the thermal storage effect of PCA and compare it with the temperature control effect of ordinary crushed stone, three parallel experiments were conducted in this study, which were full PCA, half PCA–half stone group and full stone group. To ensure the heating amount and heat dissipation boundary are consistent for the three groups of experiments at the same time, this experiment used self-made water bath heating equipment. Add 500 mL EPC insulation particles at the top of each water bath liquid level to provide top insulation, as shown in Figure 11. In this study, temperature sensors and collection instruments were used to monitor and collect the temperature changes between the three groups of aggregates during the process of heating the water bath to 85 °C. The experimental equipment is shown in Figure 12.

## 4. Test Procedure of PCAC

Based on the equal volume method, the PCA granules prepared above were used to replace the coarse aggregates in concrete, and a total of four sets of mixed proportions of concrete were designed. They are the experimental control group with a replacement rate of 0% for coarse aggregates (S1), the unencapsulated PCA group with 100% replacement for the coarse aggregates group (W1), the superfine cement-encapsulated PCA group with 100% replacement for coarse aggregates group (C1), and the epoxy resin-encapsulated PCA group with 100% replacement for the coarse aggregate group (H1). The specific mix proportions are shown in Table 5.

According to the Standard for test methods of concrete physical and mechanical properties (GB/T50081–2019) [46], experiments were conducted on the compressive, flexural and splitting tensile properties of PCAC.

## 5. Results and Discussion

### 5.1. Mechanical Properties of PCA

#### 5.1.1. Cylindrical Compress Strength of PCA

The results of the phase-change aggregates cylindrical compressive strength test are shown in Table 6. It can be seen from Table 6 that the order of cylindrical compressive strength of the tested materials is lightweight ceramsites < unencapsulated PCA < superfine cement-encapsulated PCA < epoxy resin-encapsulated PCA. Comparing the compressive strength of lightweight ceramsites with that of unencapsulated PCA, it can be seen that the adsorption of phase change materials can improve the compressive strength of the aggregates. When the adsorption ratio is 81.34%, the compressive strength increment of the aggregates is 29%. Compared with unencapsulated PCA, superfine cement-encapsulated PCA and epoxy resin-encapsulated PCA, it can be seen that both superfine cement shell and epoxy resin shell can increase the strength of the aggregates, with epoxy resin increasing it by more than superfine cement. Superfine cement-encapsulated PCA increased its compressive strength by 0.5%, while epoxy resin-encapsulated phase change aggregates increased its compressive strength by 8.7%.

**Figure 13:**
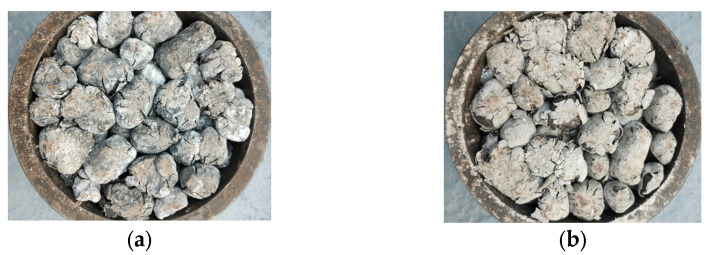
The failure mode of the PCA after cylindrical compress strength test. (**a**) Epoxy resin-encapsulated PCA; (**b**) Superfine cement-encapsulated PCA.

**Figure 14:**
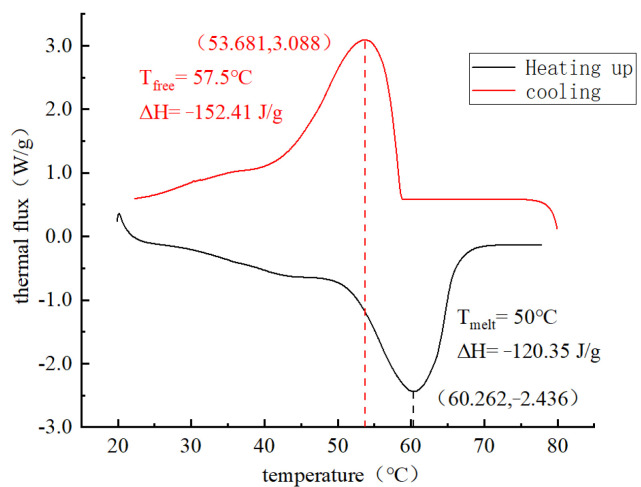
The DSC curves of paraffin.

The failure mode of the epoxy resin-encapsulated PCA (Figure 13a) and superfine cement-encapsulated PCA (Figure 13b) cylinder compressive strength specimens is shown in Figure 14. The degree of shell breakage of the PCA encapsulated with epoxy resin is smaller than that of the phase change aggregates encapsulated with superfine cement slurry, indicating that the strengthening effect of epoxy resin on the PCA is better than that of superfine cement slurry encapsulation.

#### 5.1.2. Crushing Rate of PCA during Mixing

After stirring in a forced mixer for 240 s, separate the PCA from the coarse aggregates, observe the fragmentation of PCA shells which were made of superfine cement and epoxy resin and calculate the shell crushing rate. The calculation results are shown in Table 7. According to the results, the crushing rate of the shell made of superfine cement is 42%, while the crushing rate of the shell made of epoxy resin is 0. So superfine cement slurry is not the preferred material for encapsulating PCA, while epoxy resin has a significant protective effect on PCA.

### 5.2. Thermodynamic Properties of PCA

#### 5.2.1. Enthalpy Value of PCA

**Figure 15:**
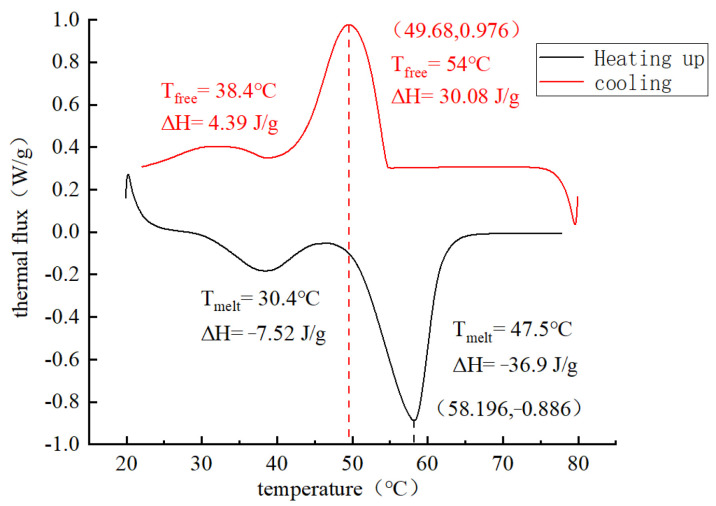
The DSC curves of phase change aggregates.

**Figure 16:**
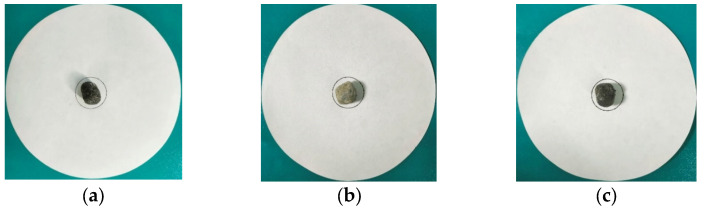
Aggregates before thermal stability tests. (**a**) Unencapsulated PCA; (**b**) PCA encapsulated in superfine cement; (**c**) PCA encapsulated in epoxy resin.

The DSC curves of paraffin melting and crystallization are shown in Figure 14. It can be seen from Figure 14 that the peak melting temperature of paraffin is around 60 °C, which is very close to its phase change peak temperature of 58 °C, indicating that the test results are accurate and reliable. The peak melting and crystallization temperature of paraffin is only one, indicating that the paraffin used in this experiment has high purity. The DSC curves of melting and crystallization of PCA are shown in Figure 15. It can be seen from Figure 16 that there are two peak temperatures for the melting and crystallization of PCA. The temperature peak with a higher absolute value of enthalpy for the melting and crystallization of paraffin, and the melting peak temperature of paraffin, is 58 °C. The temperature peak with a lower absolute value of enthalpy is the melting and crystallization of a certain component in PCA.

#### 5.2.2. Thermal Stability of PCA

**Figure 17:**
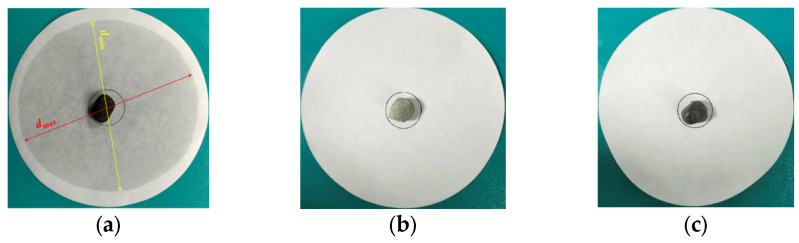
Aggregates after thermal stability tests. (**a**) Unencapsulated PCA; (**b**) PCA encapsulated in superfine cement; (**c**) PCA encapsulated in epoxy resin.

Figure 16 and Figure 17 show the images of the unencapsulated PCA, superfine cement-encapsulated PCA and epoxy resin-encapsulated PCA before and after thermal stability tests, respectively. The unencapsulated PCA granules have obvious flowing-out of PCM as is shown in Figure 16a and Figure 17a. From Figure 16b,c and Figure 17b,c, it can be seen that the PCA encapsulated with superfine cement and epoxy resin have no signs of phase change material flowing out. Based on the above analysis, the results are listed in Table 8. Based on the above analysis and combined with the evaluation results of PCA thermal stability in the literature [41,47], the thermal stability of phase change aggregates encapsulated in superfine cement and epoxy resin is really stable.

#### 5.2.3. Thermal Storage Performance of PCA

**Figure 18:**
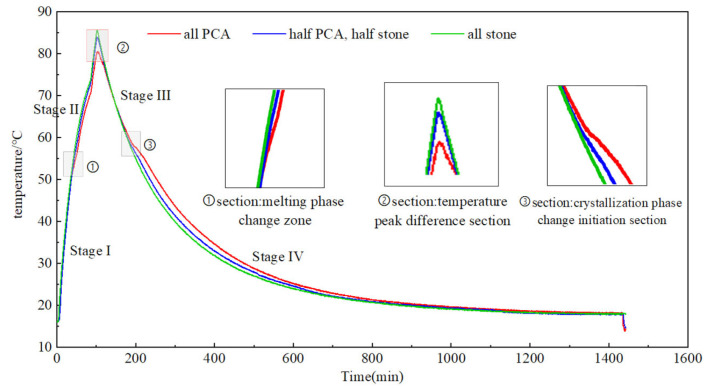
Heat storage and release curve of phase change aggregates under semi-adiabatic temperature rise condition.

Figure 18 shows the heat storage and release curves of PCA under semi-adiabatic temperature rise conditions. The curves are divided into the initial stage of the temperature rise stage (stage I), melting phase change section (section ①), phase change temperature rise stage (stage II), temperature peak difference section (section ②), amorphous temperature drop stage (stage III), crystallization phase change initiation section (section ③) and crystallization phase change stage (stage IV).

The initial stage of temperature rise section (stage I): During this stage, the temperature rise curves of the three samples are consistent, and there is no deviation of the temperature curve. Based on the DSC curves of the PCA mentioned above, it can be seen that the initial phase change temperature of the PCM in the PCA is about 50 °C. Therefore, the main reason for the lack of deviation of the temperature curve at this stage is that the melting phase change of the PCM in the PCA has not occurred.

Melting phase change zone (section ①): As shown in Figure 18, the PCA begins to phase change in this zone, and the temperature curves of the full PCA group and half PCA-half stone group begin to deviate. The temperature curve deviation of the full PCA group is greater than that of the half PCA–¬half stone group, and the deviation temperature is about 52 °C, which is very close to the phase change temperature of the phase change material in the DSC curves.

Phase change temperature rise section (stage II): After passing through the melting phase change zone, the temperature curves of various groups continue to deviate. As the temperature rises, the temperature will continue to increase according to the deviation curve, and the degree of deviation will increase with the increase in temperature.

Temperature peak difference zone (section ②): Observe the peak temperature of the full stone group. When the peak temperature of the full stone group reaches 85 °C, stop heating and turn off the heater power. In this zone, it can be clearly observed that the full stone group has the highest peak temperature, followed by the peak temperature of the half stone–half PCA group, and the full PCA group has the lowest peak temperature. The three temperature peaks are 85.37 °C, 83.92 °C and 80.21 °C, respectively, indicating that phase change materials have a significant effect on temperature control. Moreover, the full stone group reached the peak temperature 6 min earlier than that of the full PCA group, which also indicates phase change aggregate has a delay effect in temperature control.

Amorphous temperature drop section (stage III): At this stage, the PCM inside the PCA is in a liquid melting state, and there is no significant deviation in the temperature drop curves of the three groups.

Crystallization phase change initiation zone (section ③): As the temperature decreases, when the initial crystallization temperature of the PCM inside the PCA is reached, the PCM begins to transform from liquid to solid, and the PCM will release heat. Compared with the DSC curves of phase change aggregates, the initial crystallization temperature of the phase change aggregates is about 53 °C. The results of this experiment show that the initial crystallization phase change zone occurs at around 57 °C.

Crystallization phase change section (stage IV): During the cooling stage, the phase change materials in the phase change aggregates gradually begin to crystallize and release heat. With the increase in time, the temperature drop curves of the three sets of tests finally overlap.

By summarizing the effect of PCA heat storage performance on temperature control in literature [28,35,39,42], it can be seen that the influence of PCA on temperature field includes two aspects: peak clipping effect and delay effect. In the results of this study, the peak clipping effect of PCA on temperature is relatively significant, and it is also accompanied by a certain delay effect, which is similar to the results of previous studies.

### 5.3. Mechanical Properties of PCAC

#### 5.3.1. Compressive Performance

**Figure 19:**
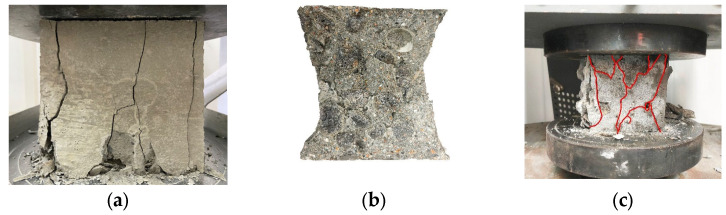
Fracture section of concrete after compressive test. (**a**) Ordinary concrete; (**b**) Encapsulated PCAC; (**c**) Unencapsulated PCAC.

Figure 19 shows the failure test blocks of different coarse aggregates in compressive tests, in which all the failure test blocks are similar in shape, and a residual failure main block can still be seen after the failure. With the increase in load on unencapsulated PCAC (Figure 19c), small irregular cracks appear in the entire section. As the load continues to be applied, the width and depth of the cracks increase, and the test block finally appears as a loose body. The main reason is that there is a thin layer of paraffin on the surface of the unencapsulated PCA, there is a gap in the combination with the cement-based material and the PCA cannot be combined with the cement matrix. On the contrary, a tight bond between the PCA and the cement slurry is achieved after the PCA has been encapsulated with ultra-fine cement or epoxy resin. According to the failure surface, there is no trace of coarse aggregate fracture on the failure surface of ordinary crushed stone concrete, but PCA fracture on the failure surface of PCAC. The results show that the strength of PCA is not higher than that of crushed stone and cement matrix.

**Figure 20:**
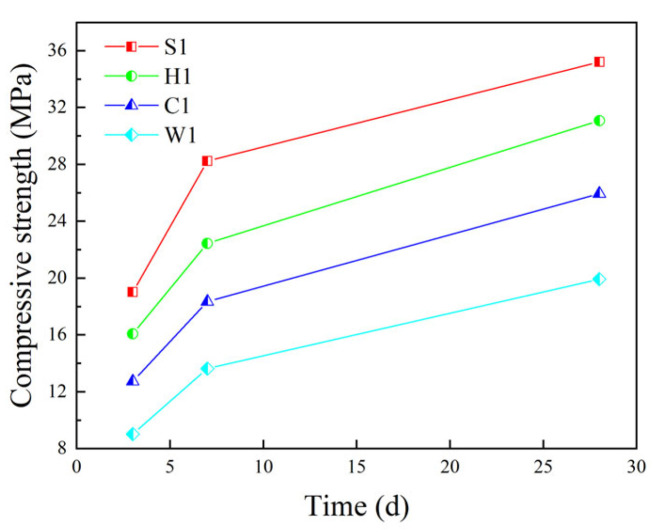
Compressive strength of concrete with different coarse aggregates.

**Figure 21:**
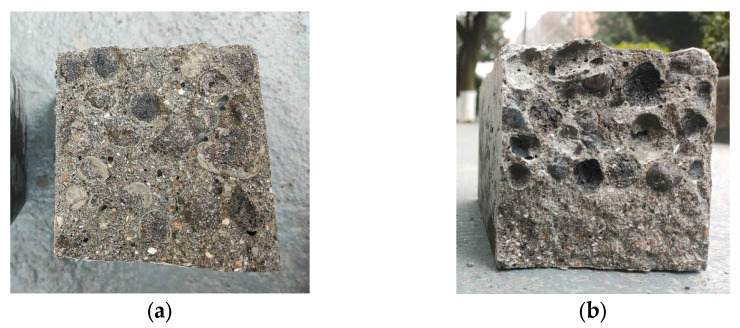
Fracture section of concrete after flexural test. (**a**) Unencapsulated concrete; (**b**) Encapsulated concrete.

Figure 20 shows comparisons of the compressive strength of different coarse aggregate concrete. It can be seen from Figure 21 that for the 3d, 7d and 28d compressive strength of concrete, ordinary crushed stone concrete (S1) > epoxy resin-encapsulated PCAC (H1) > superfine cement-encapsulated PCAC (C1) > unencapsulated PCAC (W1).

As the curing age of concrete increases, the compressive strength of both PCAC and ordinary crushed stone concrete increases. However, the reduction percentage between the compressive strength of PCAC and that of ordinary crushed stone concrete varies less with the increase in curing time. For unencapsulated PCAC, the compressive strength of the concrete compared to ordinary crushed stone concrete at 3d, 7d and 28d is reduced by 52.5%, 51.72% and 46.2%. The compressive strength is reduced by 34.4%, 35% and 30.0% for superfine cement-encapsulated PCAC. The compressive strength is reduced by 15.6%, 20.5% and 19.2% for epoxy resin-encapsulated PCAC. In addition, the strength growth rate of ordinary concrete in the early stage is faster than that of PCAC, and there is little difference in the later stage.

#### 5.3.2. Flexural Performance

Figure 21 shows the fracture sections of different coarse aggregate concrete in the flexural test. It can be seen from the fracture sections of the buckling test that the PCA is broken on all unencapsulated PCAC sections, while only PCA cavities exist on the encapsulated PCAC sections, indicating that the failure of unencapsulated PCAC is due to insufficient PCA strength, while the failure of encapsulated PCAC is caused by cracking at the connection surface between the PCA and the cement base because the bond strength between the PCA and the cement base is lower than the strength of the PCA.

**Figure 22:**
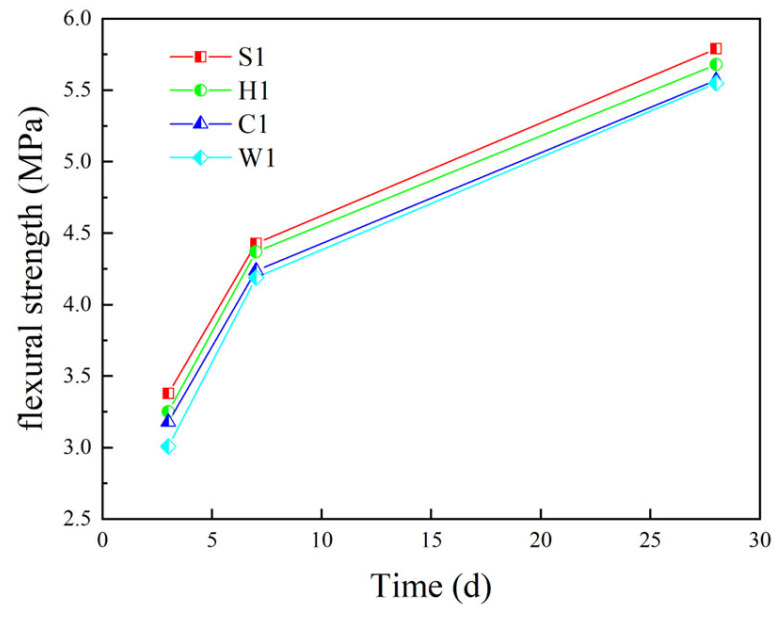
Flexural strength of concrete with different coarse aggregates.

Figure 22 shows the variation in the flexural strength of different coarse aggregate concretes. It can be seen from Figure 22 that the flexural strength of different coarse aggregate concretes is ordinary crushed stone concrete (S1) > epoxy resin-encapsulated PCAC (H1) > superfine cement-encapsulated PCAC (C1) > unencapsulated PCAC (W1). Epoxy resin-encapsulated PCAC has 0.11 MPa, 0.26 MPa and 0.31 MPa lower flexural strength in 3d, 7d and 28d compared to ordinary crushed stone concrete. Superfine cement-encapsulated PCAC has 0.21 MPa, 0.37 MPa and 0.48 MPa lower flexural strength in 3d, 7d and 28d. Unencapsulated PCAC has 0.31 MPa, 0.51 MPa and 0.80 MPa lower flexural strength in 3d, 7d and 28d. The difference in flexural strength between ordinary concrete and PCAC is not large, and the strength growth rate is similar.

#### 5.3.3. Splitting Tensile Performance

**Figure 23:**
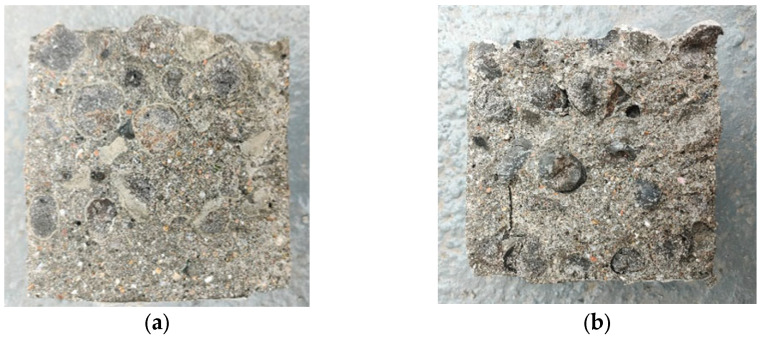
Fracture section of concrete after splitting tensile test. (**a**) Unencapsulated concrete; (**b**) Encapsulated concrete.

Figure 23 shows the comparison of fracture sections in the splitting tensile test of different coarse aggregate concretes. It can be seen from the fracture sections that the fracture tensile failure mode of different aggregate concretes is similar to the fracture failure mode. The unencapsulated PCAC aggregate is damaged, while the connection surface of encapsulated PCAC is damaged.

**Figure 24:**
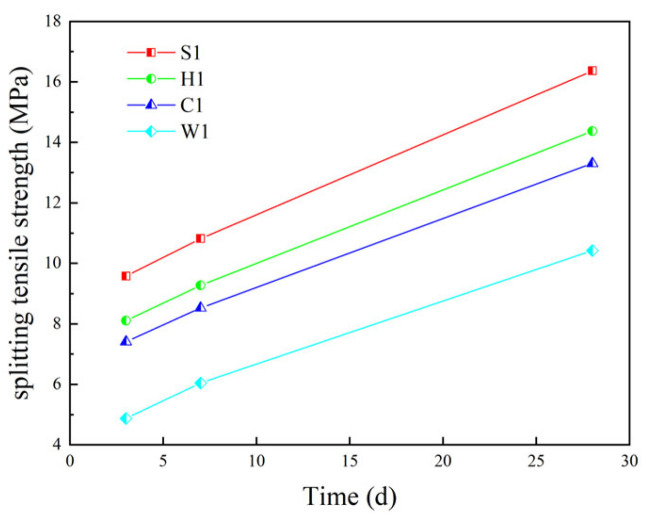
Splitting tensile strength of concrete with different coarse aggregates.

Figure 24 shows the variation of splitting tensile strength of different coarse aggregate concrete. It can be seen from Figure 24 that the order of splitting tensile strength of different coarse aggregate concrete is ordinary crushed stone concrete (S1) > epoxy resin-encapsulated PCAC (H1) > superfine cement-encapsulated PCAC (C1) > unencapsulated PCAC (W1). When replacing all the coarse aggregates in concrete with PCA, the splitting tensile strength decreases. After the PCA is encapsulated, the splitting tensile strength increases compared to the unencapsulated PCA. The 3d, 7d and 28d splitting tensile strength of epoxy resin-encapsulated PCAC is reduced by 15.3%, 14.2% and 12.2% compared to ordinary crushed stone concrete. The 3d, 7d and 28d splitting tensile strength of superfine cement-encapsulated PCAC decreased by 22.7%, 21.2% and 18.7%. The results show that compared to the superfine cement-encapsulated PCAC, epoxy resin-encapsulated PCAC has higher splitting tensile strength.

The mechanical properties of PCAC are summarized in Table 9. By comprehensive comparison of the mechanical properties of PCAC and ordinary concrete, the mechanical properties of PCAC have been reduced to varying degrees, which has been reflected in previous studies. Hunger et al. [48]. and Fernandes et al. [49] have found that the mechanical properties of concrete with PCM decrease mainly in two aspects. The first is that the PCA itself has lower strength and greater brittleness. The second is damage and destruction of the PCA during the mixing, leading to flowing out of the phase change material, thereby damaging the hydration reaction and reducing the mechanical properties. Therefore, damage of the shell and the flowing out of phase change materials lead to a decrease in the connection performance between the cement matrix and the aggregates, which is an important reason for the decrease in the mechanical properties of concrete.

In practical engineering, the defect of strength reduction caused by phase change aggregate can be made up by appropriate improvement of concrete grade, because the cost increase caused by appropriate improvement of concrete grade is far smaller than that caused by concrete cracking due to hydration heat.

### 5.4. Control Effect of the Hydration Heat of PCAC

Considering factors such as site constraints in the laboratory, it is difficult to place mass concrete in the laboratory. Therefore, this paper designs an equivalent water bath heating test, based on the law of conservation of energy, to equivalently replace the heat of PCA in a water bath environment into concrete, so as to investigate the effect of heat storage of phase change aggregates on the temperature control of the hydration reaction of mass concrete.

The heat storage energy of phase change aggregates can be calculated according to Equation (4):

(4) $$Q_{P}=c_{w}m_{w}\left(t_{2}-t_{1}\right)$$

where *Q_P_* is the heat storage of phase change aggregate, *c_w_* is the specific heat capacity of water, *m_w_* is the quality of test water, *t*_2_ is the water temperature when adding phase change aggregates and *t*_1_ is the water temperature when adding crushed stone.

The heat storage capacity of phase change aggregate per unit mass can be calculated according to Equation (5):

(5) $$q_{P}=\frac{Q_{P}}{m_{P}}$$

The heat storage capacity of mass concrete with a phase change aggregate content of *m* can be calculated by Equation (6):

(6) $$Q_{c}=q_{P}m$$

The relationship between the heat storage of phase change aggregates in phase change concrete and temperature is:

(7) $$\;Q_{c}=cm_{c}\left(T_{2}-T_{1}\right)$$

The specific heat capacity of phase change concrete is:

(8) $$\;c=c_{pc}n_{c}+c_{ps}n_{s}+c_{pw}n_{w}+c_{pb}n_{b}$$

From Equations (4)–(8), it can be found that:

(9) $$\;c_{w}m_{w}\left(t_{2}-t_{1}\right)m=cm_{c}\left(T_{2}-T_{1}\right)m_{P}$$

where *m_P_* is the quality of phase change aggregate added in the experiment, *T*_2_ is phase change concrete temperature, *T*_1_ is the temperature of ordinary crushed stone concrete, *c_pc_* is the cement specific heat capacity, *n_c_* is the ratio of cement mass to concrete mass, *c_ps_* is the heat capacity of fine aggregate, *n_s_* is the ratio of fine aggregate mass to concrete mass, *c_pw_* is the specific heat capacity of water, *n_w_* is the ratio of water mass to concrete mass, *c_pb_* is the specific heat capacity of phase change material and *n_b_* is the ratio of phase change material mass to concrete mass.

**Figure 25:**
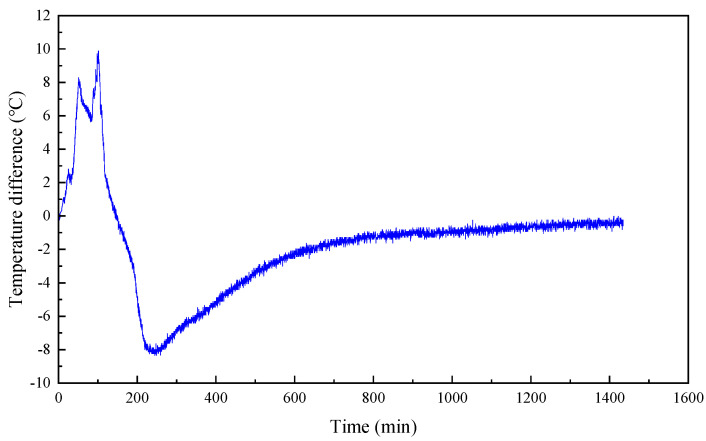
Temperature curve of phase change aggregate concrete and ordinary crushed stone concrete under semi-adiabatic condition.

Based on the above derivation, the experimental results are converted into the temperature curve of phase change concrete and ordinary crushed stone concrete at the same volume of phase change aggregate. The conversion relationship is based on Equation (8), and the conversion parameters are shown in Table 10. The conversion results are shown in Figure 25. As can be seen from Figure 25, the temperature difference between phase-change aggregate concrete and ordinary concrete can reach 10 °C at most, and the temperature control effect is relatively ideal. In fact, the temperature control effect of phase change materials obtained by simulating the release of hydration heat in concrete by water bath heating in this experiment is conservative, because the actual concrete hydration reaction occurs around each phase change aggregate, and the heat of phase change aggregate is more direct and uniform than that of water bath heating, so the actual concrete temperature control effect of phase change aggregate in this study should be better than the theoretical calculation.

## 6. Conclusions

This research prepares PCA, and selects different encapsulating materials to encapsulate the prepared PCA. Research was conducted on the mechanical properties of the PCA and the thermal storage effect of PCAC. The conclusions are as follows:

(1) Both superfine cement and epoxy resin can be used as preferred materials for PCA encapsulation and shell making. When encapsulating PCA with epoxy resin, graphite powder can be added to the epoxy resin slurry to increase its thermal conductivity.
(2) Superfine cement shell and epoxy resin shell can increase aggregate strength, with epoxy resin having a better effect than superfine cement. The PCAs encapsulated in superfine cement and in epoxy resin both have good thermal stability.
(3) For the concrete at 3d, 7d and 28d, the mechanical properties of concrete mixed with PCA are reduced to varying degrees compared to ordinary crushed stone concrete.
(4) When the water bath temperature reaches 85 °C, the temperature difference between the PCA and the common stone aggregate can be up to 6 °C, and based on the law of conservation of energy, the test results will be converted to mass concrete with the same volume of the aggregate mixture, The difference of PCAC and ordinary concrete temperature can be up to 10 °C, and the temperature control effect is significant.

## Figures and Tables

**Table 1:** Main performance indexes of ceramic granules.

Ceramic Granules	Particle Size	Cylinder Compression Strength	Water Absorption	Bulk Density
Lightweight black ceramic granules	5–30 mm	16.99 MPa	9%	753 kg/m^3^

**Table 2:** Main performance indexes of No. 58 fully refined paraffin.

Shape	Phase Change Temperature Peak	Density	Specific Heat	Latent Heat of Phase Transition
White block	58 °C	900 kg/m^3^	2.25 kJ·kg^−1^·K^−1^	225.1 J·g^−1^

**Table 3:** Mechanical properties of superfine cement.

Performance Index	3 Days Compressive Strength	28 Days Compressive Strength	3 Days Flexural Strength	28 Days Flexural Strength
Value	≥30 MPa	≥60 MPa	≥5 MPa	≥7 MPa

**Table 4:** Parameters of component A of epoxy resin.

Performance Index	Chroma	Epoxy Equivalent	Hydrolyzed Chlorine	Inorganic Chlorine	Volatile Matter	Softening Point
Value	≤Pt-Co	≤210~230 g/mol	≤0.50%	≤50 mg/kg	≤0.60%	14~23 °C

**Table 5:** Specific parameters of concrete mix proportions.

No.	Replacement Rate of Coarse Aggregate (%)	Cement (kg/m^3^)	Sand (kg/m^3^)	Crushed Stone (kg/m^3^)	Phase Change Aggregate (kg/m^3^)	Water (kg/m^3^)	Water Reducing Agent (kg/m^3^)
S1	0%	295	760	1024	0	160	85
W1	100%	295	760	0	805	160	85
C1	100%	295	760	0	825	160	85
H1	100%	295	760	0	782	160	85

**Table 6:** The results of the PCA cylindrical compressive strength test.

Material	Mean Value of Cylindrical Compressive Strength (MPa)	Standard Deviation (MPa)
Lightweight ceramsites	16.99	0.12
Unencapsulated PCA	21.95	0.21
Superfine cement-encapsulated PCA	22.06	0.21
Epoxy resin-encapsulated PCA	23.85	0.39

**Table 7:** Results of crushing rate of phase change aggregates during mixing.

Encapsulating Material	Superfine Cement Slurry	Epoxy Resin
Crushing rate	42%	0

**Table 8:** The results of thermal stability of PCA.

	Unencapsulated Phase Change Aggregates	Phase Change Aggregates Encapsulated in Superfine Cement	Phase Change Aggregates Encapsulated in Epoxy Resin
$d_{max}$ (mm)	141	0	0
$d_{min}$ (mm)	157	0	0
$d_{mean}$ (mm)	149	0	0
Percentage of exudation	397	0	0

**Table 9:** The summary of mechanical properties of PCAC.

The Mechanical Properties of PCAC(MPa)						
Superfine Cement-Encapsulated				Epoxy Resin-Encapsulated		
	3 d	7 d	28 d	3 d	7 d	28 d
Compressive performance	12.74 (1.03)	18.35 (1.54)	25.95 (1.89)	16.08 (0.67)	22.45 (0.97)	31.09 (1.24)
Flexural performance	3.18 (0.23)	4.24 (0.34)	5.57 (0.66)	2.76 (0.32)	4.37 (0.27)	5.68 (0.46)
Splitting tensile performance	7.41 (0.45)	8.53 (0.57)	13.31 (0.36)	8.11 (0.56)	9.28 (0.78)	14.38 (1.09)

Remark: The numbers in parentheses are standard deviations.

**Table 10:** Conversion parameters.

Parameters	*c_w_*/(kJ·kg^−1^·K^−1^)	*m_w_*/(kg)	*m*/(kg)	*c*/(kJ·kg^−1^·K^−1^)	*m*_c_/(kg)	*m_p_*/(kg)
Value	4.2	8	3.03	3.2	5.64	3.03

## Data Availability

Not applicable.
